# Seawater cooling of PV modules mounted on ships in Świnoujście/Poland harbour^☆^

**DOI:** 10.1016/j.heliyon.2022.e10078

**Published:** 2022-08-10

**Authors:** Zbigniew Zapałowicz, Wojciech Zeńczak

**Affiliations:** a Department of Energy Technologies, Faculty of Mechanical Engineering and Mechatronics, West Pomeranin University of Technology, Szczecin Al. Piastów 17, 70-310, Poland; b Department of Safety and Energy Engineering, Faculty of Maritime Technology and Transport, West Pomeranian University of Technology, Szczecin Al. Piastów 17, 70-310 Szczecin, Poland

**Keywords:** PV system on ships, Ship power system, Cooling PV modules

## Abstract

New international marine regulations concerning sea transport indicate that one of the ways to meet them is the use of renewable energy sources (RES) on ships. The PV installation mounted on ships is one of solutions. Cooling of PV modules is a way to improve their capacity. The paper presents results of calculation on capacity of PV modules mounted on ships where seawater is used as cooling agent. The calculations were carried out for conditions prevailing in the harbour Świnoujście/Poland. The aim of this paper is to compare PV module's power gain in six characteristic months (January to June) of the statistic year. Analysis of the results obtained for Świnoujście shows that application of seawater in cooling systems of PV modules on ships is justified only for spring and summer seasons.

## Highlights

• PV installation on ships.
• Cooling of PV module by means of seawater.
• Improvement of PV module power efficiency by water cooling.

## 1 Introduction

One of the key problems of modern world are climate changes created by expansive human activity. The changes result, among others, from the combustion of fossil fuels in order to obtain energy of various kinds. Meaningful energy consumer is transport. The energy is crucial to drive units or vehicles, but on the other side, the environment suffers from high level of atmospheric emissions in the process of energy production. It is therefore very important to effectively use the obtained energy and thus diminish its consumption in various ways: using the recovery energy, replacement of fossil fuels by alternative fuels, e.g. biofuels, or the energy from renewable sources. The issue is particularly significant in case of ships, where additional problems appear, namely possibilities of bunkering and application of some alternative fuels. International Maritime Organization (IMO) and European Union introduce progressively more restrictive legislation which forces designers and ship-owners to search for new solutions of ship's propulsion systems [1]. According to IMO, emission of CO_2_ in transport should be reduced by at least in 40% a short-term time perspective till 2030 as related to 2008. In further perspective, that is till 2050, the emission should be reduced by 70%. On the other hand, reduction of total gas emission in international shipping should amount at least 50% in the further time perspective. Strategy of IMO assumes zero GHG emission in 2100 [2,3].

Solar energy is one of RES that can be used to obtain electric power on ships [4, 5, 6]. In this technology, the basic element of PV installation, the PV module, is power converter. However, the ship's area to be used for the PV installation is quite limited and so the amount of generated power. Kobougias et al. in their work [4] presented PV cells and installations that are currently used on ships and gave the advantages and disadvantages of the proposed solutions. The concept of seawater cooling of PV modules was first presented by Konur and Erginer [6]. Experimental tests carried out on a PV module made independently by these researchers, showed an increase in its efficiency from 1.5 to 5%, when cooled by seawater near the port of Izmir/Turkey. Thus, PV installation can only provide the basic power on the ship [7, 8]. Large ocean-going ships have propulsion powers ranging from several to several dozen megawatts, however, despite the relatively large available area of 2.000 to even 5.000 m^2^, the installation of photovoltaic panels on such ships cannot cover the entire power demand. PV systems are highly depending on the accessibility of solar radiation. Besides, they require short-term storage systems for the generated power. Because of extremely difficult working conditions (humidity, salinity, strong winds, flooding by high waves, etc.), production of PV installations requires special technologies and materials, which in turn, elevates costs of investments. Another impairment is small efficiency of energy conversion. It is about 20% for working conditions of standard PV modules. Of course, there are modules with higher efficiencies, still, their production costs are meaningfully higher because of their more sophisticated structures [9]. Also systems that track the sun are not worthy their price because of high costs, e.g. need for movable parts and for additional power to drive the tracking device.

One of ways to improve the power efficiency of a PV module is to lower the temperature on the p-n junction by the application of a cooling system. PV modules can be cooled by natural or by forced modes [10, 11, 12, 13, 14, 15, 16, 17, 18, 19, 20, 21, 22, 23, 24, 25]. The first one is cheap but ineffective. The next one is more expensive because of the need for additional power, but it is more effective. According to authors of the paper, cooling of PV panels is the least expensive way to upgrade their efficiency. Technical solution applied for a number of years is a combination of PV module with traditional liquid collector, that is, module of PVT type [26].

The present paper considers possibilities of the application of seawater as a working agent in cooling installations of PVT modules mounted on ships. Authors accomplished initial analysis for an installation of the above type which could be mounted on any ship, e.g. passenger-vehicle ferry [27]. Calculations of power gain were made for 12 advised days of the year, for a vessel that berthed in the harbor of Świnoujście/Poland, or in the vicinity, e.g. on its roadstead. According to conclusions in paper [27], the inconvenient season to use cooling systems in PV modules is winter, and the better time is late spring. However, more detailed analysis of PV installation's operation should focus on power increase for each statistic day of the month. The aim of paper is the comparison of the PV module's power gains for each day of six characteristic months (January to June) of a typical statistic year. The obtained results of PV module power increment calculations for each day of the month will allow a better evaluation of the considered cooling system solution than data for a single day. The reason for undertaking the research is that there is relatively little information available in the world bibliography concerning the proposed PV module cooling system solution (Konur and Erginer's research [6]).

## 2 Research methodology

### 2.1 Assumptions

PV installation consisted of a system of PV modules mounted on flat, unshaded surfaces (decks, upper structures) of a given ship. The ship was berthed in the harbor of Świnoujście/Polska, or in waters near the harbor. The ship disposed of closed circuit of cooling freshwater, and its efficiency was sufficient to collect the heat from the cooling system of PV modules.

**Figure 1:**
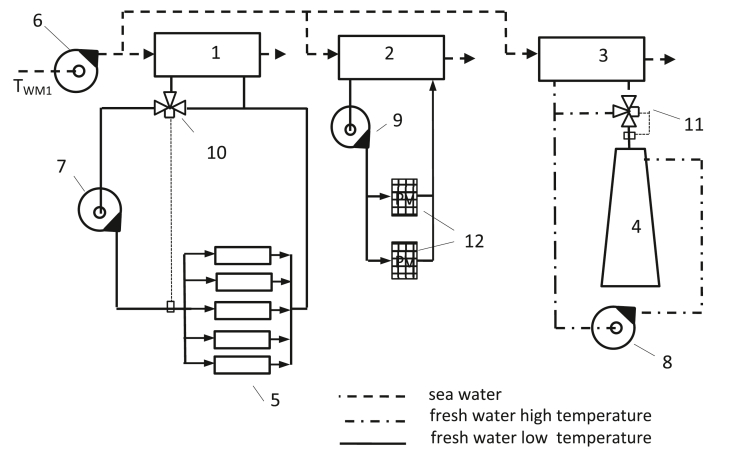
Simplified scheme of the central cooling system of a power plant using an additional independent central cooler of a low-temperature freshwater circuit for cooling of PV panels. 1-low-temperature system central cooler; 2-PV panel cooling fresh water cooler; 3-high-temperature system central cooler; 4-main and auxiliary engines; 5-engine room equipment and media requiring cooling; 6-cooling overboard water pump; 7-low-temperature system cooling fresh water pump; 8-high-temperature system cooling fresh water; 9-cooling fresh water pump in the PV panel cooling system; 10-low-temperature system thermostatic valve; 11-high-temperature system thermostatic valve; 12-PV panel cooling fresh water cooler.

Figure 1 shows a simplified scheme of the central cooling system of the power plant using an additional independent central cooler of the low-temperature freshwater circuit for cooling the PV panels. The use of a central cooling system avoids direct cooling by aggressive seawater.

Calculations were made for the best working conditions of PV installations operation. Each PV module was cooled with freshwater the temperature of which equalled the temperature of outboard water (temperature drop in intermediate heat exchanger of the cooling system being neglected). Temperature increase of cooling freshwater in PV module was assumed to be constant and it equalled 0.2 °C. The assumption allowed to obtain the best cooling conditions, but it required the flow intensity of cooling water through heat exchanger being controlled. The coolant pump operating in the cooling system of PV modules had to be equipped with power inverter that enabled changes of rotation speed of the impeller. Analysis of effect of cooling the PV module onto gain or drop of its power was carried on for a single module of this installation. The cooling effect was estimated as power gain from 1m^2^ of its area.

### 2.2 Calculation methodology

The methodology of calculation is presented in paper [27]. The paper gives analysis of energy balance for PV modules without cooling ($t_{M}$) and with cooling ($t_{M\_CH}$) operated in quasi-steady state. Temperatures $t_{M}$ and $t_{M\_CH}$ for both analyzed PV modules were calculated from balance equations. It was assumed that temperatures of their p-n junctions equalled calculated temperatures of PV modules. Power gain of PV module was calculated from Eq. (1):

(1) $$\Delta P_{M}=P_{M,STC}\cdot \left(\frac{C_{P,\;t_{M}}}{100}\right)\cdot \left(G/1000\right)\cdot \Delta t_{M},$$

where $P_{M,STC}$ - nominal power, $C_{P,\;t_{M}}$- coefficient of power change with temperature, G - total solar irradiance, $\Delta t_{M}$ - PV module temperature drop.

Temperatures difference of modules without and with cooling was calculated from Eq. (2):

(2) $$\Delta t_{M}=\;t_{M}-t_{M\_CH}$$

### 2.3 Data to calculations

Monocrystalline PV module of STP320S-20/Wfh type, offered by SELFA, was chosen for the analysis. Technical data of single PV module needed for calculations are given in Table 1. It should be added that the PV module analyzed is used in land-based solutions. In maritime solutions, it should be further noted that the PV module will operate in a more aggressive environment (seawater flooding, salt deposition, etc.).

**Table 1:** Technical data of PV module of STP320S-20/Wfh type [28].

No	Parameter	Symbol	Unit	Value
1	Power (STC)	P_M,STC_	W	320
2	Power coefficient	C_P,tM_	%/°C	0.4
3	Surface area, gross	A_M_	m^2^	1.656

Besides, it was assumed for calculations that reflectivity and emissivity of reflective layer of the PV module's surface equalled respectively r_M_ = 0.02 i ε_M_ = 0.93 [29,30]. Heat transfer coefficient between PV module and air (h_M-A_) was calculated according to recommended by ASHRAE equation which relates overflowing the module's flat surface by wind. In case of PV module with cooling system, it was assumed that heat transfer coefficient from the back wall of PV module was constant and it equalled 223 W/(m^2^K). Commonly available meteorological data [31] for the region of Świnoujście were used for calculations.

**Figure 2:**
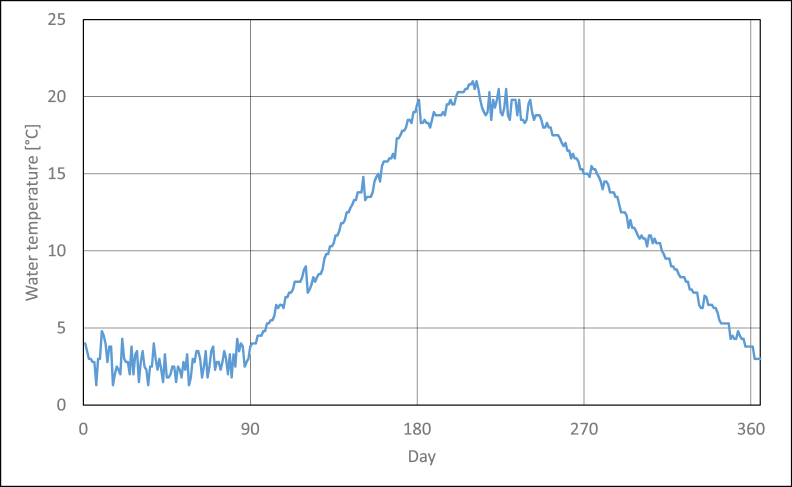
Mean seawater temperature vs successive days of year.

Figure 2 presents temperature changes of seawater in the Baltic Sea near Świnoujście. Given temperature values are mean 24-h values of seawater temperature in the years 2018–2021 [32].

According to statistic data, the temperature is nearly constant in the first three months, and its values are included in the range of 2–4 °C. In the beginning of April, 24-h temperatures of seawater begin to increase and they reach their maximum values at the turn of July and August. Seawater temperature at this time is about 20–21 °C. Then, gradual drop of seawater temperature is observed in successive months, and its value is again about 3.5 °C at the end December. If the first three months of the year are neglected, nearly symmetric water temperatures distribution in function of time is to be observed. For the above reason, detailed analysis concerning possibilities of PV modules’ cooling was restricted to only the first part of statistical year.

Also the meteorological database for Świnoujście [31] was used for calculations, that is, mean hourly values of air temperatures, wind velocities, irradiance.

## 3 Results and discussion

**Figure 3:**
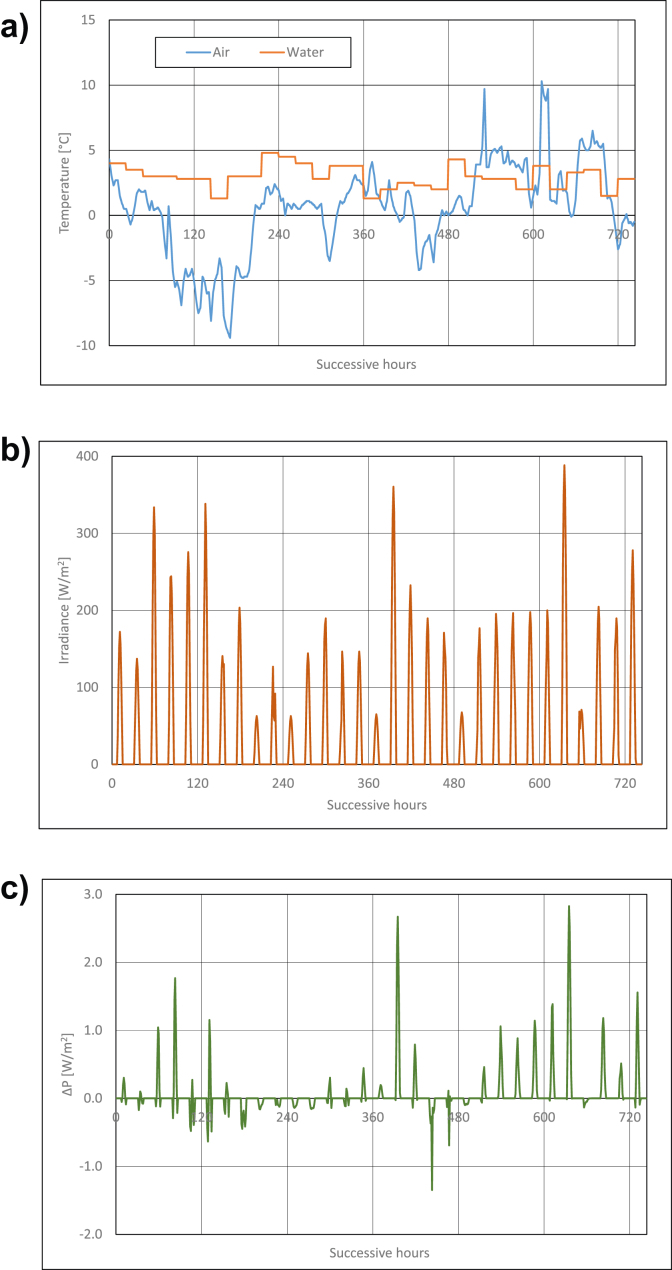
Characteristic parameters in January; (a) air and water temperatures (b) irradiance (c) gain of PV module power.

**Figure 4:**
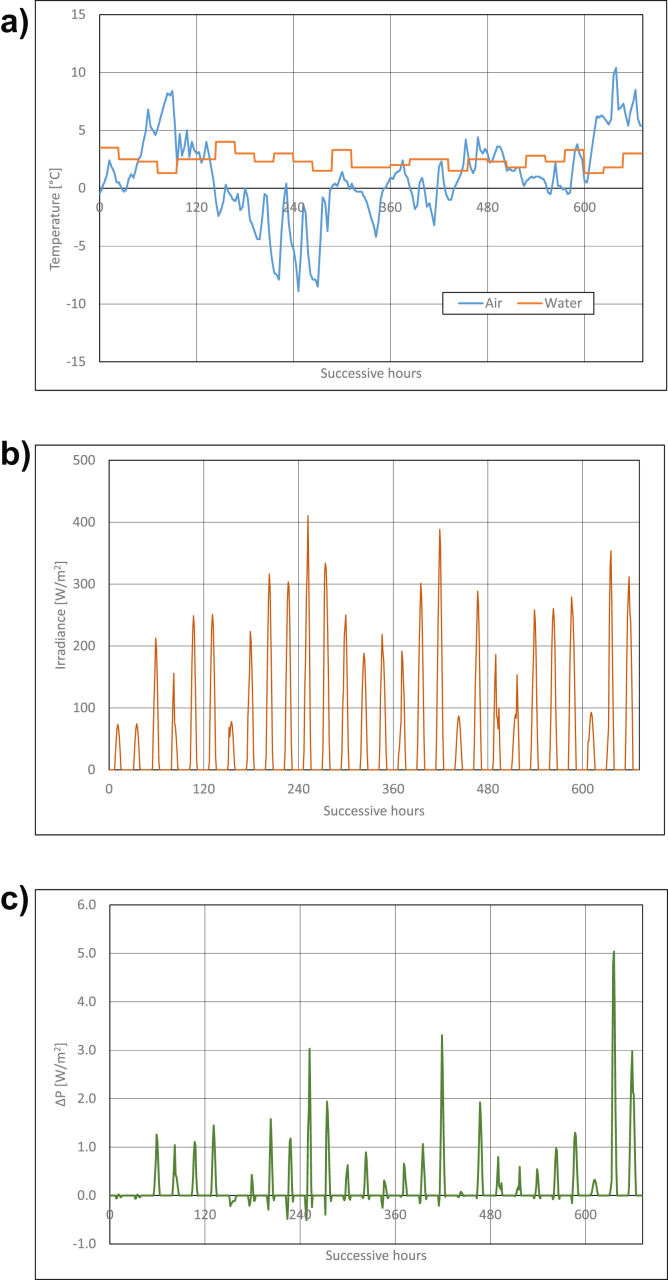
Characteristic parameters in February; (a) air and water temperatures (b) irradiance (c) gain of PV module power.

**Figure 5:**
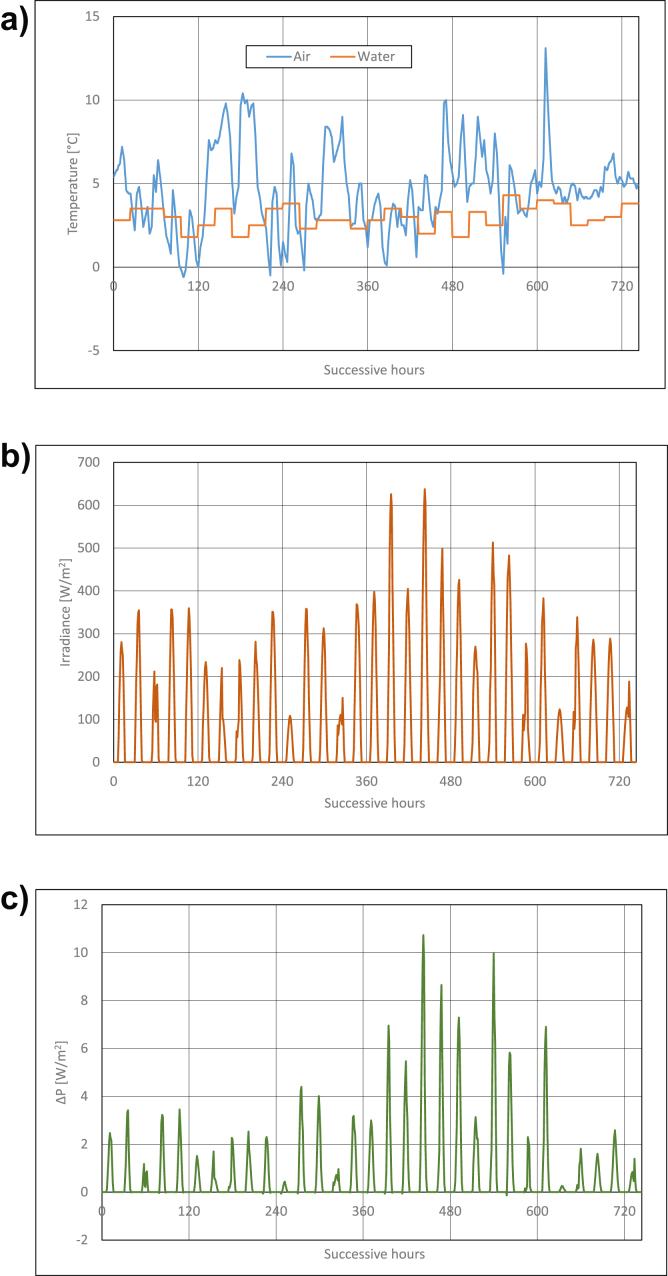
Characteristic parameters in March; (a) air and water temperatures (b) irradiance (c) gain of PV module power.

**Figure 6:**
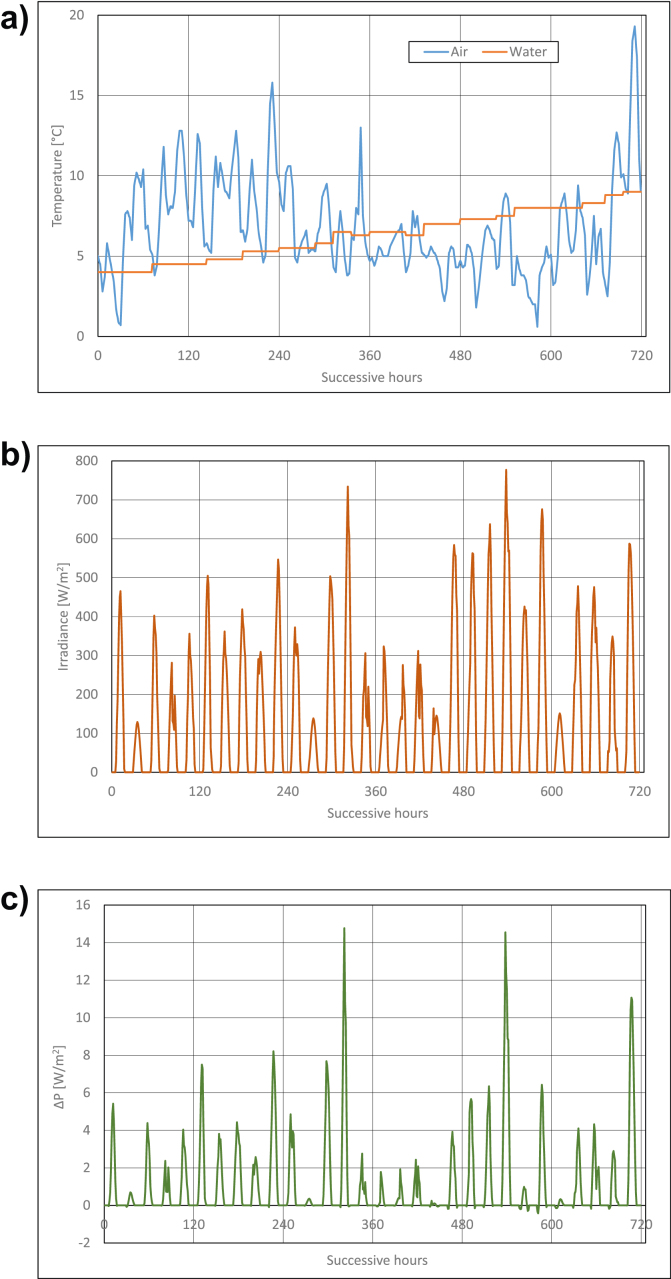
Characteristic parameters in April; (a) air and water temperatures (b) irradiance (c) gain of PV module power.

**Figure 7:**
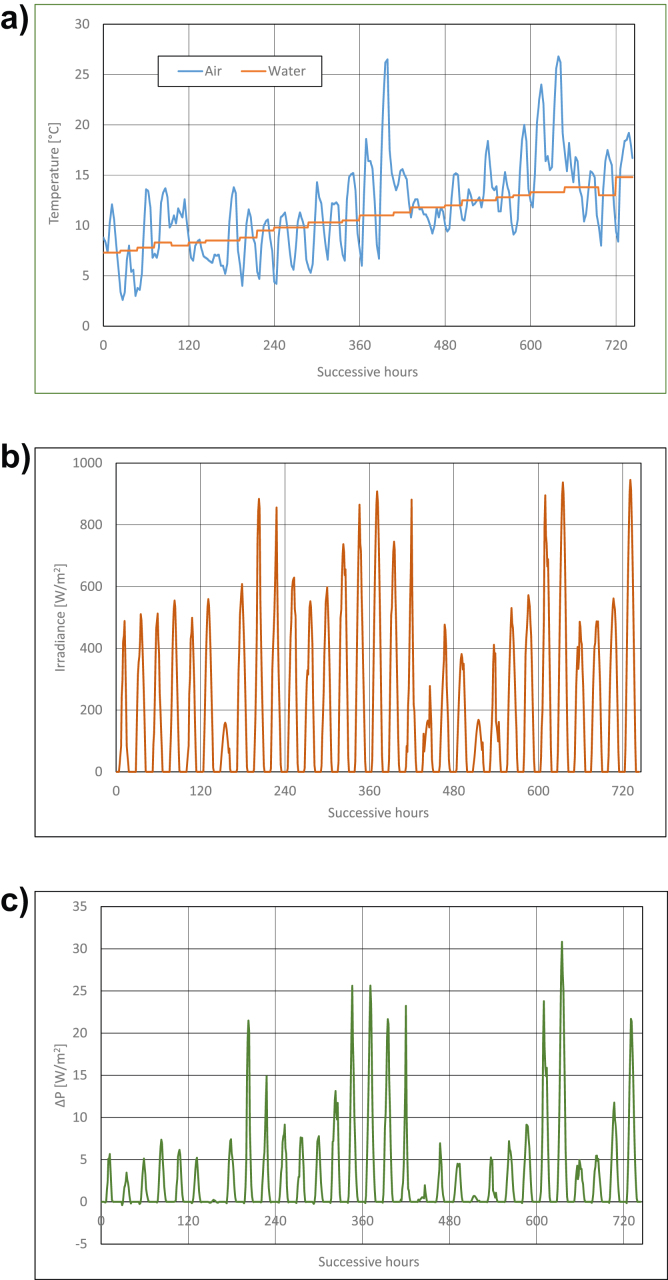
Characteristic parameters in May; (a) air and water temperatures (b) irradiance (c) gain of PV module power.

**Figure 8:**
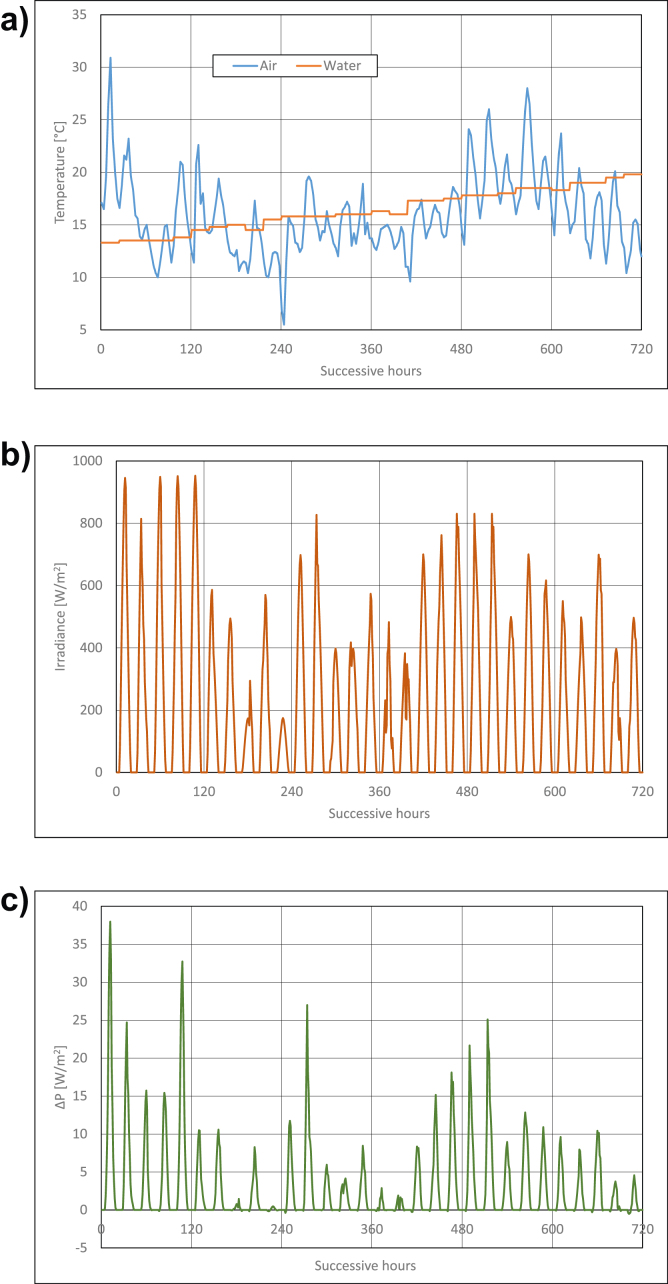
Characteristic parameters in June; (a) air and water temperatures (b) irradiance (c) gain of PV module power.

Calculations results are presented in Figures 3, 4, 5, 6, 7, and 8. For each of analyzed months, charts were drawn. They illustrate changes of temperatures of air and seawater, irradiance, and power gain of PV module in function of successive hours of the month. The data analysis applies only to hours of day, because the solar radiation occurs only in daytime and so power is generated. The cooling system shouldn't operate at night, because there is no necessity to cool the PV module.

Figure 3 presents changes of characteristic parameters in January. Twenty-four-hours temperatures of seawater in the Baltic Sea in January remains in the range from +1 °C do +5 °C (Figure 3a). Further, air temperature for this month changes in the range from -10 °C to +10 °C (Figure 3a). In the first and the second decades of the month, air temperature is lower than seawater temperature, the trend is inversely related in the third decade of the month. Irradiance in January is relatively small and it remains in the range from ca 50W/m^2^ to ca 400W/m^2^ (Figure 3b). It results from analysis of PV module's power gain presented in Figure 3c that positive values of the above parameter reach in most favourable conditions only ca 3W/m^2^ (that is below 1% of its nominal power). On some days, operating cooling system of PV module generates loss of module's power, which is caused by the fact that air temperature is lower than that of water, and irradiance is low. In this case, air is a better cooling agent than seawater which warms the PV module up. In January, the cooling system should be set in operation only when power gain of PV module is positive, that is, after the sunrise. The system should be switched off before the sunset.

Water temperature in the Baltic Sea in February is still low and its values are in the range from ca +1.5 °C to +4 °C (Figure 4a). The range of air temperatures changeability is similar to the changes in the preceding month and it is from -10 °C to +10 °C (Figure 4a). Most of hours in February, air temperature is lower than seawater temperature. Similarly, irradiance is still low and it equals from about 50W/m^2^ to about 400W/m^2^ (Figure 3b). Power gain of PV module equals on average ca 1W/m^2^, which is only 0.3% of its rated power. In most favourable conditions, the gain reached the value of 5W/m^2^. Thus, operating the cooling system of PV module in February should be like in January.

In March, water temperatures in the Baltic Sea are more levelled and they are in the range from ca +2 °C to +4 °C (Figure 5a). On the other hand, the range of air temperatures changes is smaller than in previous months and it is from ca 0 °C to +10 °C (Figure 5a), and only for one from statistical days of March this temperature is higher and it equals about +13 °C. Also changes of irradiance in March are more levelled and they are from about 100W/m^2^ to 400 W/m^2^ (Figure 4b), though irradiance can be higher for a few days and it can equal even 600 W/m^2^. By far higher are power gains of PV module, they reach maximal values of ca 10–11W/m^2^ (that is ca 3.5% of rated power) on most favourable day.

April is the month when seawater temperature grows rapidly up to ca +8 °C (Figure 6a) at the end of the month. In turn, the air temperature is positive, but very changeable. It can be higher than the seawater temperature in the first part of the month (Figure 6a). In the second part of the month, it can be lower or higher than the seawater temperature. Irradiance changes from ca 100 W/m^2^ to 800 W/m^2^. PV module power gains in most favourable conditions reach values of ca 15W/m^2^ (that is ca 4.7% of rated power of PV module).

In May, doubling of seawater temperature value is observed (Figure 7a). This temperature grows to ca +14 °C at the end of the month. Air temperatures change in wide range of values. Amplitude of changes is nearly 24 °C. Irradiance equals from ca 500W/m^2^ to 900W/m^2^ for most of days of May. These values belong to the highest ones in the year (Figure 7b). Then, some of highest values of PV module's power gains in the year occur in May (Figure 7c). In most favourable conditions, the power gain is even as high as 30W/m^2^ (that is 9.5% of rated power).

In June, seawater temperature grows and it has the value of ca +20 °C (Figure 8a) at the end of the month. Amplitude of air changes is about 24 °C (Figure 8a). Irradiance exceeds the value of 900W/m^2^ (Figure 8b) on some days. Irradiance values lower than 400W/m^2^ occur only on a few days of June. The highest power gain of PV module is to be stated in June. It equals ca 38W/m^2^ (that is 11.8% of rated power) (Figure 8c). However, on some days of June, PV module power gain can also be very insign-ificant.

## 4 Conclusions

On the basis of analysis of results of carried out research, it can be stated that:

a) application of seawater as a working agent in cooling systems of PV modules mounted on ships is possible, but detailed technical, economic, and ecological analysis is required,
b) cooling system of PV modules should operate periodically, depending on mutual relations among temperatures of cooling seawater and of air, and irradiance,
c) in the analyzed calculation case, for waters near the harbour of Świnoujście, the best power gains of cooled PV module can be obtained in May and June; in this period, irradiance is very intensive, which causes that temperatures of air and of p-n junction grow, by much slower changes of seawater temperature.
d) in winter months, that is in January and February, the proposed cooling system of PV modules is not as much effective; the fact is caused by relatively small irradiance and unfavourable relations between temperatures of air and seawater,
e) the highest power gain of PV module was obtained in the beginning of June and it equalled ca 37W/m^2^, that is about 11.6% of its power in STC conditions.

## Declarations

### Author contribution statement

Zbigniew Zapałowicz: Conceived and designed the experiments; Performed the experiments; Analyzed and interpreted the data; Contributed reagents, materials, analysis tools or data; Wrote the paper.

Wojciech Zeńczak: Analyzed and interpreted the data; Contributed reagents, materials, analysis tools or data; Wrote the paper.

### Funding statement

This research did not receive any specific grant from funding agencies in the public, commercial, or not-for-profit sectors.

### Data availability statement

Data will be made available on request.

### Declaration of interests statement

The authors declare no conflict of interest.

### Additional information

No additional information is available for this paper.
